# Supplementary material for: The development of the adult nervous system in the annelid Owenia fusiformis
Source: Neural Dev. 2024 Feb 21;19:3. doi: 10.1186/s13064-024-00180-8 (PMC10880339; doi:10.1186/s13064-024-00180-8)
Supplement: Supplementary file 1 — Additional file 1: Supplementary Fig. 1. Alignment of the neuropeptide precursors P. dumerilii [30–32], C. teleta [62] and Owenia fusiformis [29]. Representative mature peptides and conserved dipeptides are highlighted in red and bold, respectively. [file 13064_2024_180_MOESM1_ESM.docx]

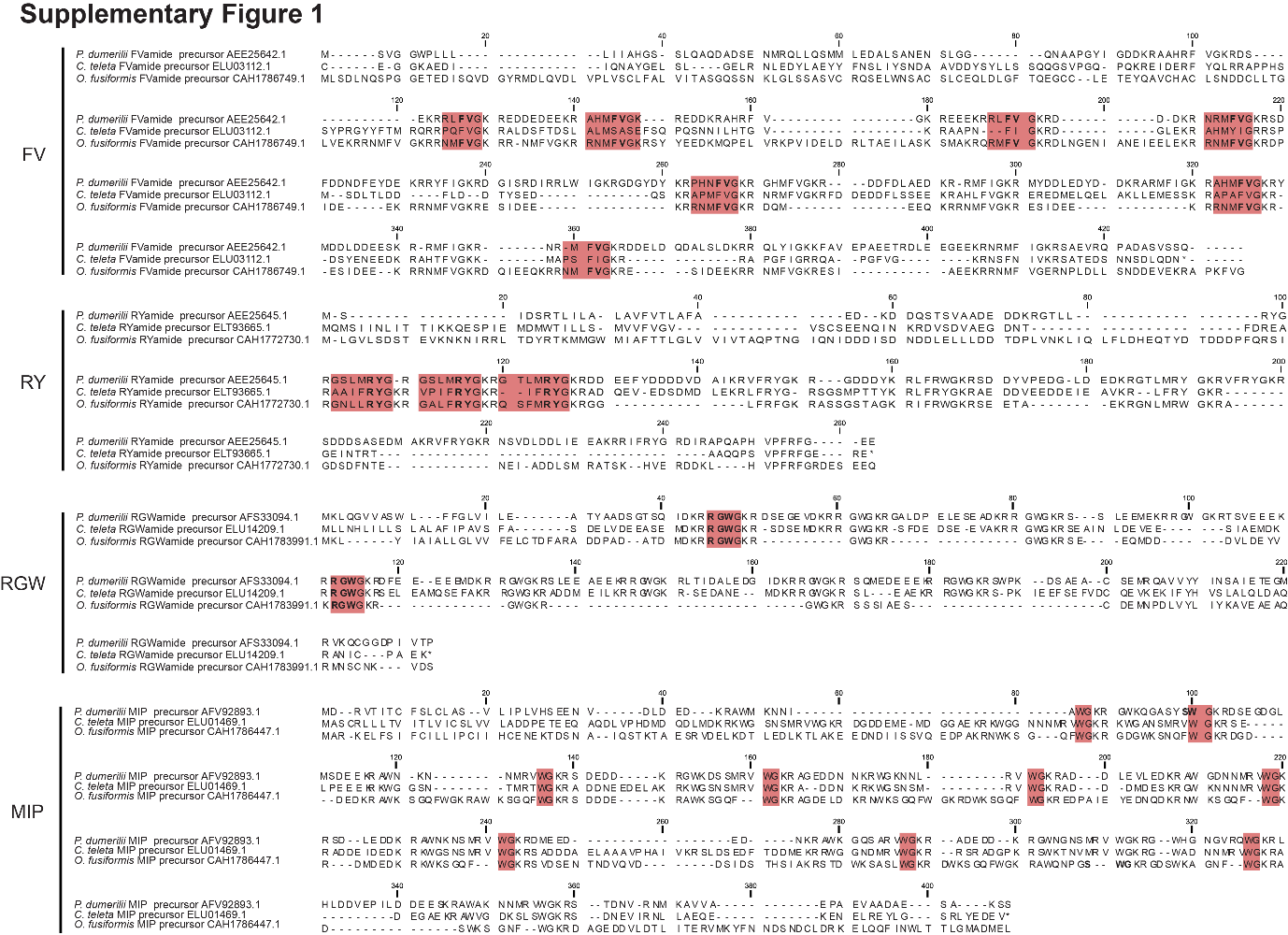
**Supplementary Information**

Additional File1: Supplementary Figure 1 Alignment of the neuropeptide precursors *P. dumerilii* (30-32), *C. teleta* (62) and *Owenia fusiformis* (29). Representative mature peptides and conserved dipeptides are highlighted in red and bold, respectively.
