# Supplementary material for: The development of the adult nervous system in the annelid Owenia fusiformis
Source: Neural Dev. 2024 Feb 21;19:3. doi: 10.1186/s13064-024-00180-8 (PMC10880339; doi:10.1186/s13064-024-00180-8)
Supplement: Supplementary file 2 — Additional file 2: Supplementary Fig. 2. MIP-lir elements in the 24hpf mitraria. MIP-lir cells include several cells as part of the apical organ (ao) and one cell anterior to the foregut (white arrow), including a MIP-lir frontal nerve (fn). Inset in b is a close up of the apical organ (ao) in the same view as the larger image. ao: apical organ; at: apical tuft; cs: chaetal sac; fn: frontal nerve; mo: mouth. [file 13064_2024_180_MOESM2_ESM.docx]

**
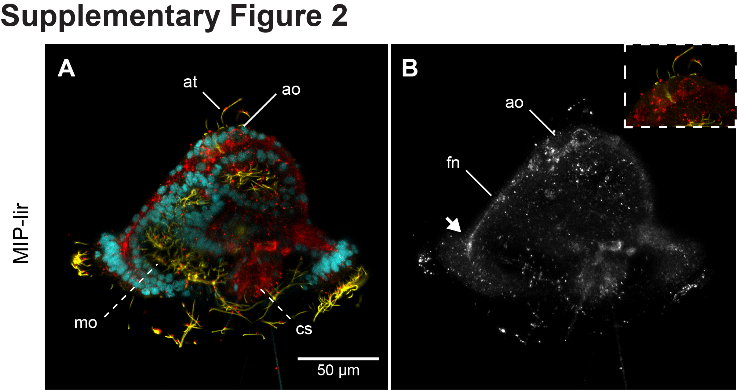
**

Additional File 2: Supplementary Figure 2 MIP-lir elements in the 24hpf mitraria. MIP-lir cells include several cells as part of the apical organ (ao) and one cell anterior to the foregut (white arrow), including a MIP-lir frontal nerve (fn). Inset in **b** is a close up of the apical organ (ao) in the same view as the larger image. ao: apical organ; at: apical tuft; cs: chaetal sac; fn: frontal nerve; mo: mouth.
