# Supplementary material for: The development of the adult nervous system in the annelid Owenia fusiformis
Source: Neural Dev. 2024 Feb 21;19:3. doi: 10.1186/s13064-024-00180-8 (PMC10880339; doi:10.1186/s13064-024-00180-8)

Additional File 3: Supplementary Figure 3. Neuropeptide-lir elements in the competent larvae. CLSM images of DAPI (cyan), acetylated tubulin (yellow) and neuropeptide-lir (red or white) elements in the competent larvae (~ 3 wpf). Lateral views, with anterior to the left. **c**, **f**, **i**, **l** are close ups of the juvenile rudiment in the same view as the respective larger image in **b**, **e**, **h**, **k**. **a–c** FVamide-lir cells and MIP-lir cells in the apical organ connect via FVamide-lir and MIP-lir circumesophageal connectives (cc) to the ventral nerve cord (vnc) of the juvenile trunk rudiment (jr), and via **a–b** FVamide-lir, **d–e** RYamide-lir and  **j–k** MIP-lir frontal (fn), dorsal (dn) and peripheral nerves (closed orange arrow heads) to the **a–c** FVamide-lir, **d–f** RYamide-lir and **j–l** MIP-lir prototrochal ring (pr). See also Figure 2. **d–f** RYamide-lir and **j–l** MIP-lir peripheral nerves also branch out to the chaetal nerve (chn) (open pink arrowheads). The foregut is innervated by **a–c** FVamide-lir and **d–f** RYamide-lir cells and neurites. By this stage the juvenile rudiment has a vnc and a **a–c** FVamide-lir and **d–f** RYamide-lir dorsal nerve cord (dnc). **g–i** RGWamide-lir cells are only present in the apical organ. **j–l** MIP-lir is present in the anterior part of the foregut (white arrow). an: anus; ao: apical organ; at: apical tuft; br: brain; cc: circumesophageal connectives; chn: chaetal sac nerve; cs: chaetal sac; dn: dorsal nerve; dnc: dorsal nerve cord; fg: foregut; fgn: foregut nerve; fn: frontal nerve; jr: juvenile rudiment; mg: midgut; mo: mouth; pr: prototrochal ring; pt: prototroch; vnc: ventral nerve cord.


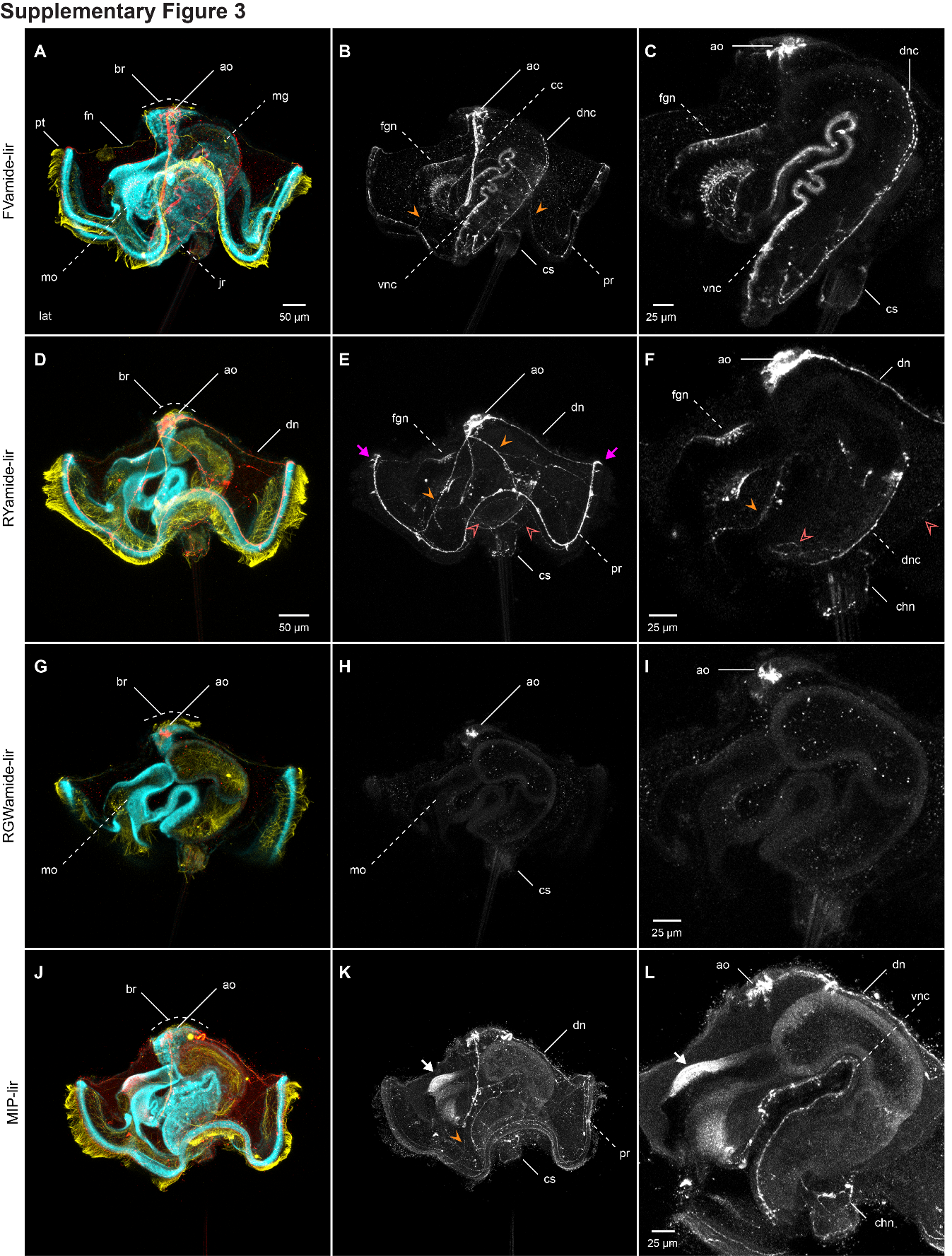

Supplement: Supplementary file 3 — Additional file 3: Supplementary Fig. 3. Neuropeptide-lir elements in the competent larvae. CLSM images of DAPI (cyan), acetylated tubulin (yellow) and neuropeptide-lir (red or white) elements in the competent larvae (~ 3 wpf). Lateral views, with anterior to the left. c, f, i, l are close ups of the juvenile rudiment in the same view as the respective larger image in b, e, h, k. a–c FVamide-lir cells and MIP-lir cells in the apical organ connect via FVamide-lir and MIP-lir circumesophageal connectives (cc) to the ventral nerve cord (vnc) of the juvenile trunk rudiment (jr), and via a–b FVamide-lir, d–e RYamide-lir and j–k MIP-lir frontal (fn), dorsal (dn) and peripheral nerves (closed orange arrow heads) to the a–c FVamide-lir, d–f RYamide-lir and j–l MIP-lir prototrochal ring (pr). See also Fig. 2. d–f RYamide-lir and j–l MIP-lir peripheral nerves also branch out to the chaetal nerve (chn) (open pink arrowheads). The foregut is innervated by a–c FVamide-lir and d–f RYamide-lir cells and neurites. By this stage the juvenile rudiment has a vnc and a a–c FVamide-lir and d–f RYamide-lir dorsal nerve cord (dnc). g–i RGWamide-lir cells are only present in the apical organ. j–l MIP-lir is present in the anterior part of the foregut (white arrow). an: anus; ao: apical organ; at: apical tuft; br: brain; cc: circumesophageal connectives; chn: chaetal sac nerve; cs: chaetal sac; dn: dorsal nerve; dnc: dorsal nerve cord; fg: foregut; fgn: foregut nerve; fn: frontal nerve; jr: juvenile rudiment; mg: midgut; mo: mouth; pr: prototrochal ring; pt: prototroch; vnc: ventral nerve cord. [file 13064_2024_180_MOESM3_ESM.docx]
