# Supplementary material for: The development of the adult nervous system in the annelid Owenia fusiformis
Source: Neural Dev. 2024 Feb 21;19:3. doi: 10.1186/s13064-024-00180-8 (PMC10880339; doi:10.1186/s13064-024-00180-8)
Supplement: Supplementary file 4 — Additional file 4: Supplementary Fig. 4. Tubulin+ elements in the competent. CLSM images of beta-tubulin (a–e) and alpha-acetylated tubulin (f–j) in the competent larvae (~ 3 wpf). a–c, g–h apical views; d–e, i–j, lateral views; f ventral view. a–c, g–h the apical organ (ao), associated with an apical tuft (at) and apical nerve ring (ar) is positioned above the brain (br). Ventral (vr) and dorsal (dr) roots c, h make the neuropil of the brain, that connects with the d cirucomesophageal connectives (cc), and ultimately with the ventral nerve cord (vnc) d–e. f, i–j Tubulin+ peripheral nerves (fn, dn, and orange arrowheads) connect the apical organ with the prototroch ring (pr). ao: apical organ; an: anus; ar: apical nerve ring; at: apical tuft; br: brain; cb: chaetoblast; cc: circumesophageal connectives; chn: chaetal sac nerve; cs: chaetal sac; dn: dorsal nerve; dnc: dorsal nerve cord; dr: dorsal root; fg: foregut; fgn: foregut nerve; fn: frontal nerve; jr: juvenile rudiment; mg: midgut; mo: mouth; nph: nephridia; pr: prototrochal ring; pt: prototroch; vnc: ventral nerve cord; vr: ventral root. [file 13064_2024_180_MOESM4_ESM.docx]

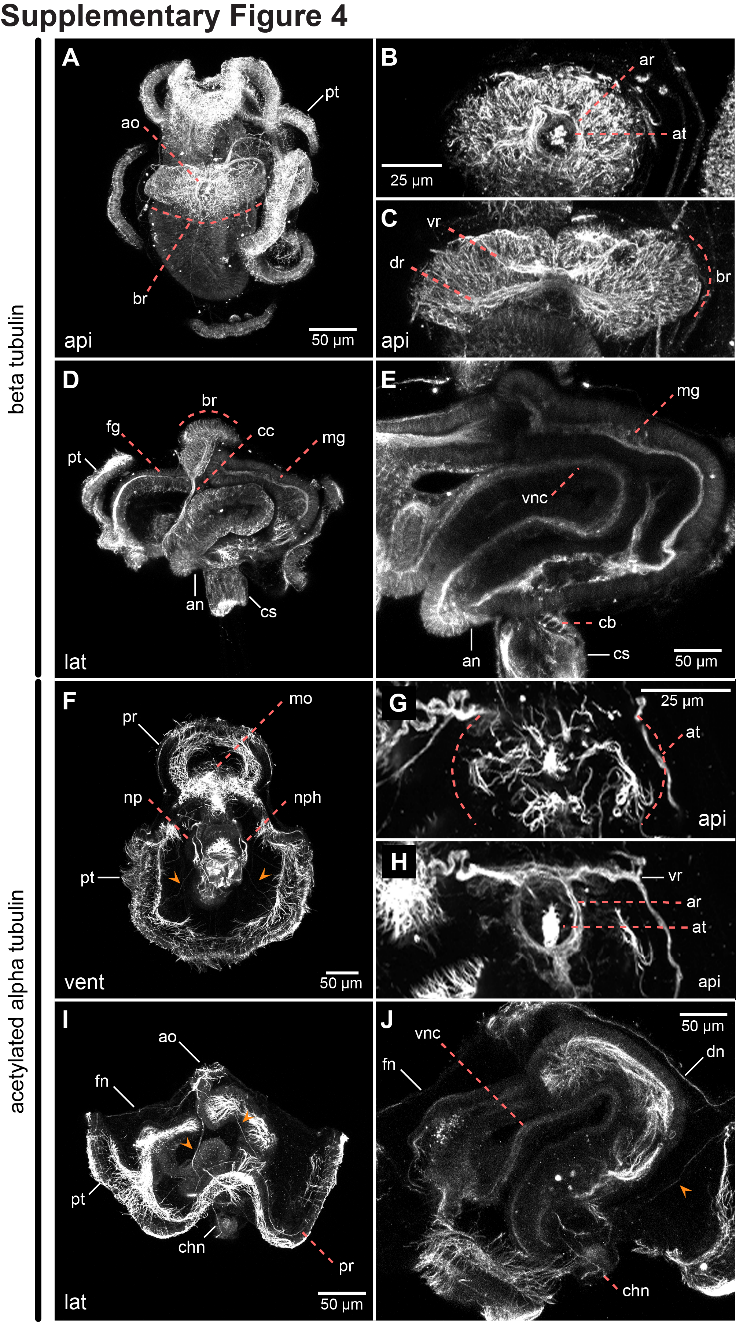
Additional File 4: Supplementary Figure 4 Tubulin^+^ elements in the competent. CLSM images of beta-tubulin (**a–e**) and alpha-acetylated tubulin (**f–j**) in the competent larvae (~3 wpf). **a–c**, **g–h** apical views; **d–e**, **i–j**, lateral views; **f** ventral view. **a–c**, **g–h** the apical organ (ao), associated with an apical tuft (at) and apical nerve ring (ar) is positioned above the brain (br). Ventral (vr) and dorsal (dr) roots **c**, **h** make the neuropil of the brain, that connects with the **d** cirucomesophageal connectives (cc), and ultimately with the ventral nerve cord (vnc) **d–e**. **f**, **i–j** Tubulin^+^ peripheral nerves (fn, dn, and orange arrowheads) connect the apical organ with the prototroch ring (pr). ao: apical organ; an: anus; ar: apical nerve ring; at: apical tuft; br: brain; cb: chaetoblast; cc: circumesophageal connectives; chn: chaetal sac nerve; cs: chaetal sac; dn: dorsal nerve; dnc: dorsal nerve cord; dr: dorsal root; fg: foregut; fgn: foregut nerve; fn: frontal nerve; jr: juvenile rudiment; mg: midgut; mo: mouth; nph: nephridia; pr: prototrochal ring; pt: prototroch; vnc: ventral nerve cord; vr: ventral root.
