# Supplementary material for: The development of the adult nervous system in the annelid Owenia fusiformis
Source: Neural Dev. 2024 Feb 21;19:3. doi: 10.1186/s13064-024-00180-8 (PMC10880339; doi:10.1186/s13064-024-00180-8)

Additional File 5: Supplementary Figure 5 SoxC orthology and early mRNA expression. **a** Maximum likelihood orthology assignments of *soxC*. **b** DIC images showing expression of *soxC* during gastrulation (9hpf) and early mitraria (24hpf). Asterisks mark the animal/apical pole an: anus; bp: blastopore; cs: chaetal sac; fg: foregut; mo: mouth; pt: prototroch.
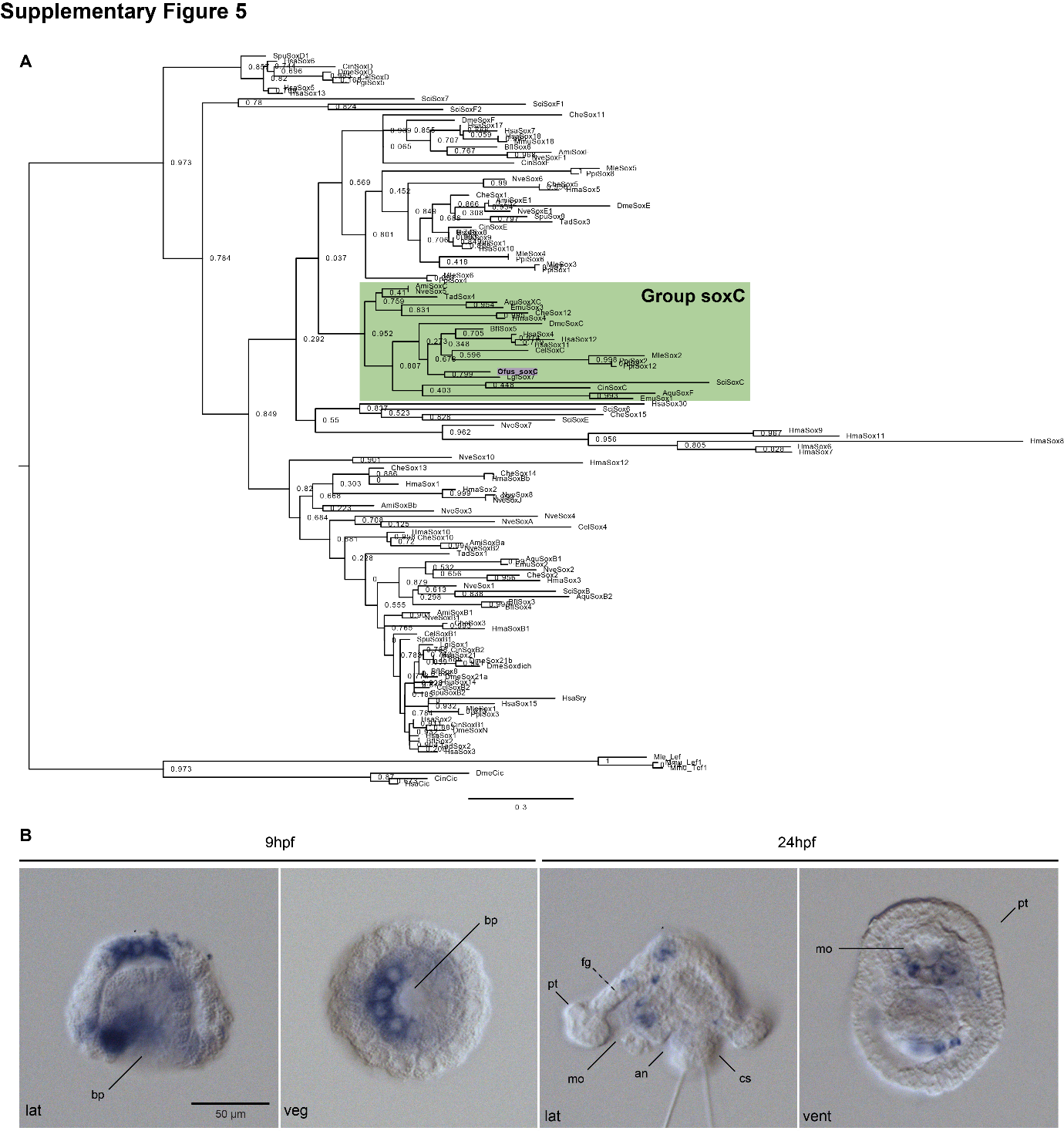

Supplement: Supplementary file 5 — Additional file 5: Supplementary Fig. 5. SoxC orthology and early mRNA expression. a Maximum likelihood orthology assignments of soxC. b DIC images showing expression of soxC during gastrulation (9hpf) and early mitraria (24hpf). Asterisks mark the animal/apical pole an: anus; bp: blastopore; cs: chaetal sac; fg: foregut; mo: mouth; pt: prototroch. [file 13064_2024_180_MOESM5_ESM.docx]
