# Supplementary material for: The development of the adult nervous system in the annelid Owenia fusiformis
Source: Neural Dev. 2024 Feb 21;19:3. doi: 10.1186/s13064-024-00180-8 (PMC10880339; doi:10.1186/s13064-024-00180-8)

Additional File 6: Supplementary Figure 6 Neuropeptide-lir elements during metamorphosis. CLSM images of DAPI (cyan) and neuropeptide-lir (red or white) elements during metamorphosis (~ 3 4pf). Lateral views, with anterior to the top. **a–h**The brain connects with the ventral nerve cord (vnc), via circumesophageal connectives (lateral medullary cords (22) at the trunk thorax, made out of three ciliated thoracis segments (cts). The foregut (fg) has **b** FVamide-lir, **f** RYamide-lir and **h** MIP-lir neurons and cells. **e–f** RWGamide labels the parapodial glandular organs (pgos), and the lower mouth lip (lml). Double yellow line marks the division between thoracic and abdominal segments. ao: apical organ; an: anus; br: brain; cc: circumesophageal connectives; cts: ciliated thoracic segments; dn: dorsal nerve; dr: dorsal root; fg: foregut; fgn: foregut nerve; lmc: lateral medullary cords; lml: lower mouth lip; ­np: brain neuropil; pgo: parapodial glandular organ 1**–**4; pr: prototrochal ring; pt: prototroch; vnc: ventral nerve cord; vr: ventral root.


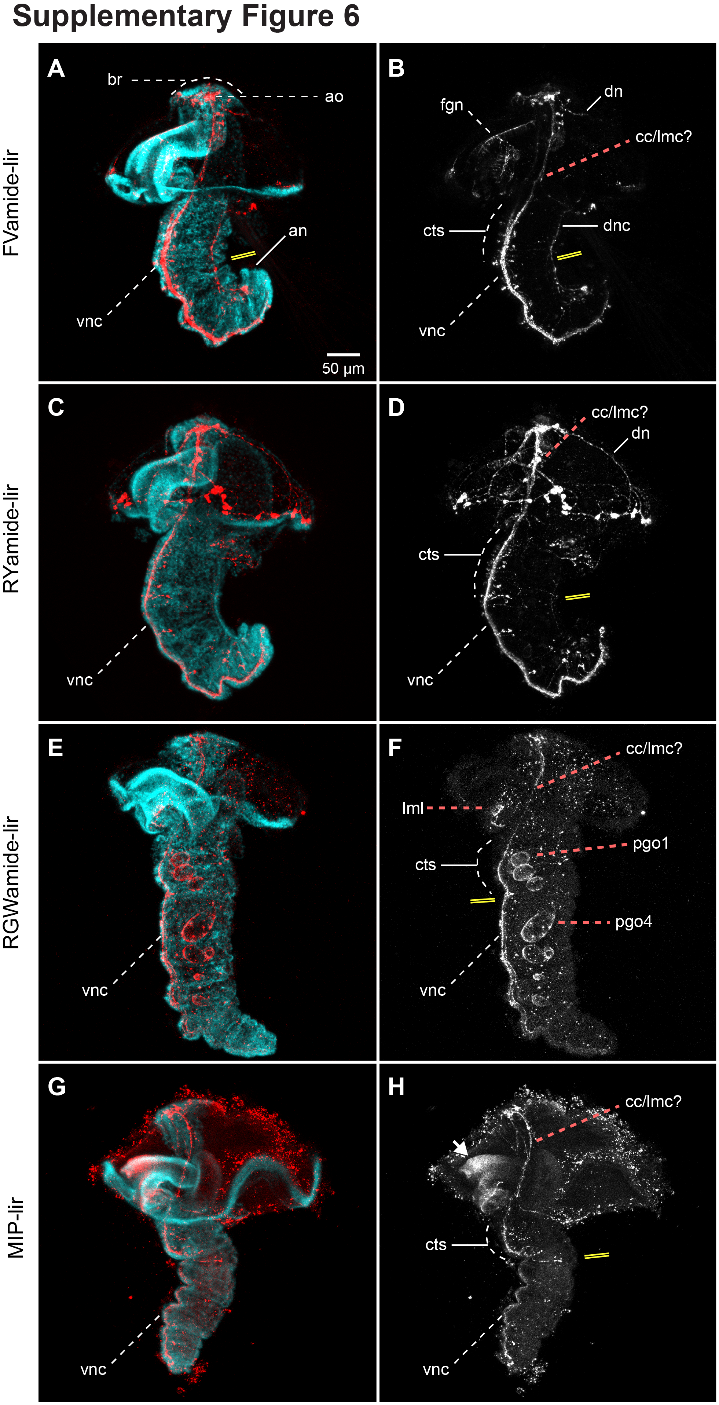

Supplement: Supplementary file 6 — Additional file 6: Supplementary Fig. 6. Neuropeptide-lir elements during metamorphosis. CLSM images of DAPI (cyan) and neuropeptide-lir (red or white) elements during metamorphosis (~ 3 4pf). Lateral views, with anterior to the top. a–hThe brain connects with the ventral nerve cord (vnc), via circumesophageal connectives (lateral medullary cords [22] at the trunk thorax, made out of three ciliated thoracis segments (cts). The foregut (fg) has b FVamide-lir, f RYamide-lir and h MIP-lir neurons and cells. e–f RWGamide labels the parapodial glandular organs (pgos), and the lower mouth lip (lml). Double yellow line marks the division between thoracic and abdominal segments. ao: apical organ; an: anus; br: brain; cc: circumesophageal connectives; cts: ciliated thoracic segments; dn: dorsal nerve; dr: dorsal root; fg: foregut; fgn: foregut nerve; lmc: lateral medullary cords; lml: lower mouth lip; np: brain neuropil; pgo: parapodial glandular organ 1–4; pr: prototrochal ring; pt: prototroch; vnc: ventral nerve cord; vr: ventral root. [file 13064_2024_180_MOESM6_ESM.docx]
