# Supplementary material for: The development of the adult nervous system in the annelid Owenia fusiformis
Source: Neural Dev. 2024 Feb 21;19:3. doi: 10.1186/s13064-024-00180-8 (PMC10880339; doi:10.1186/s13064-024-00180-8)

Additional File 7: Supplementary Figure 7 Tubulin^+^ elements during metamorphosis and juvenile. CLSM images of acetylated tubulin. **a–b** Larvae undergoing metamorphosis. **c–d** >4 wfp juvenile. **a–b** Tub^+^ peripheral nerves (orange arrowheads) in the remaining episphere of the larva keep connecting the brain to the prototrochal ring (pr). **a–d** The brain connects with the ventral nerve cord (vnc), via circumesophageal connectives (lateral medullary cords (22) at the trunk thorax, made out of three ciliated thoracis segments (cts). The vnc is composed of two robust longintudinal tracts, and two more lateral tracts (magenta arrows). On the anterior border of each segment, there is a pair of lateral transverse nerves (ln) that connect to lateral ventral-lateral longitudinal cords (magenta arrows). Double yellow line marks the division between thoracic and abdominal segments. ao: apical organ; br: brain; cc: circumesophageal connectives; cts: ciliated thoracic segments; dr: dorsal root; fn: frontarl nerve; lmc: lateral medullary cords; ln: lateral transverse nerves; nph: nephridia; pr: prototrochal ring; pt: prototroch; vnc: ventral nerve cord; vr: ventral root.


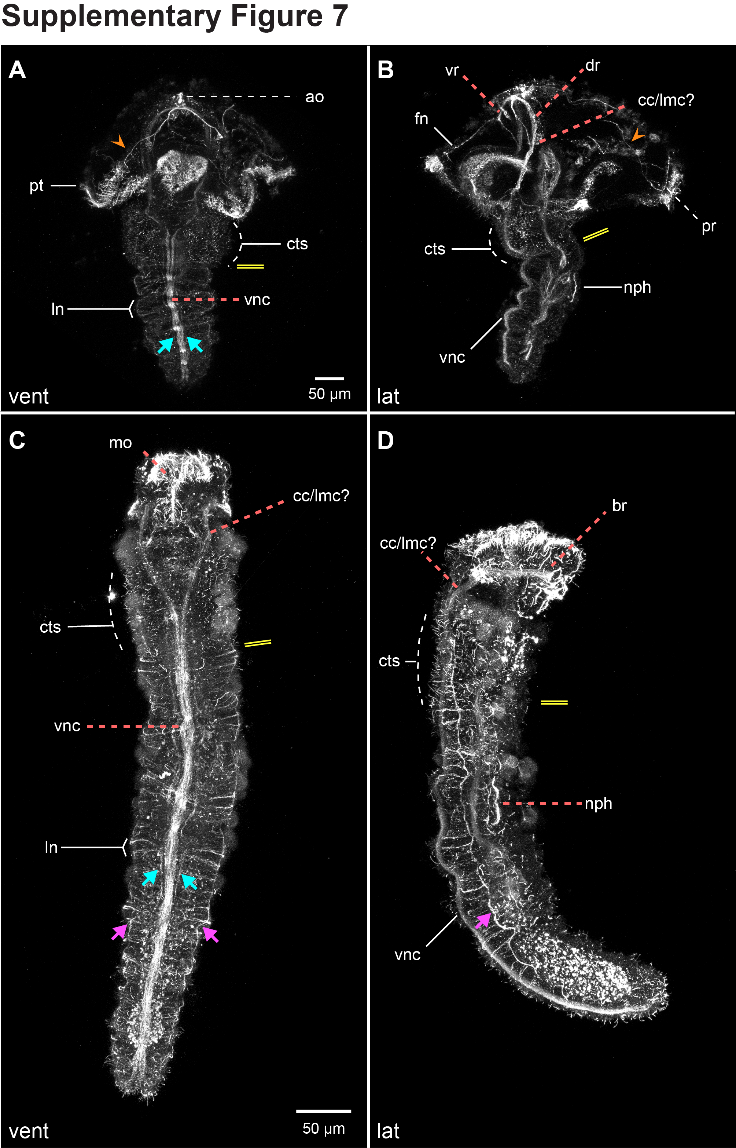

Supplement: Supplementary file 7 — Additional file 7: Supplementary Fig. 7. Tubulin+ elements during metamorphosis and juvenile. CLSM images of acetylated tubulin. a–b Larvae undergoing metamorphosis. c–d > 4 wfp juvenile. a–b Tub+ peripheral nerves (orange arrowheads) in the remaining episphere of the larva keep connecting the brain to the prototrochal ring (pr). a–d The brain connects with the ventral nerve cord (vnc), via circumesophageal connectives (lateral medullary cords [22] at the trunk thorax, made out of three ciliated thoracis segments (cts). The vnc is composed of two robust longintudinal tracts, and two more lateral tracts (magenta arrows). On the anterior border of each segment, there is a pair of lateral transverse nerves (ln) that connect to lateral ventral-lateral longitudinal cords (magenta arrows). Double yellow line marks the division between thoracic and abdominal segments. ao: apical organ; br: brain; cc: circumesophageal connectives; cts: ciliated thoracic segments; dr: dorsal root; fn: frontarl nerve; lmc: lateral medullary cords; ln: lateral transverse nerves; nph: nephridia; pr: prototrochal ring; pt: prototroch; vnc: ventral nerve cord; vr: ventral root. [file 13064_2024_180_MOESM7_ESM.docx]
