# Supplementary material for: The development of the adult nervous system in the annelid Owenia fusiformis
Source: Neural Dev. 2024 Feb 21;19:3. doi: 10.1186/s13064-024-00180-8 (PMC10880339; doi:10.1186/s13064-024-00180-8)

Additional File 8: Supplementary Figure 8 Neural development in juveniles. DIC images showing expression of *soxC*, *pou4*, *six3/6* and *otx*. **a**, **d**, **g**, **j** Lateral views; **b**, **e**, **h**, **k** ventral views; **c**, **f**, **i**, **l** dorsal views. **a–c** *soxC* and **d–f** *six3/6* are expressed in the brain (br). **g–i** *pou 4* and **j–l** *otx* have no longer any neural expression. **a–c** *soxC* is expressed in the foregut (fg), and in the putative growth zone (gz). br: brain; fg: foregut; gz: growth zone; mo: mouth.


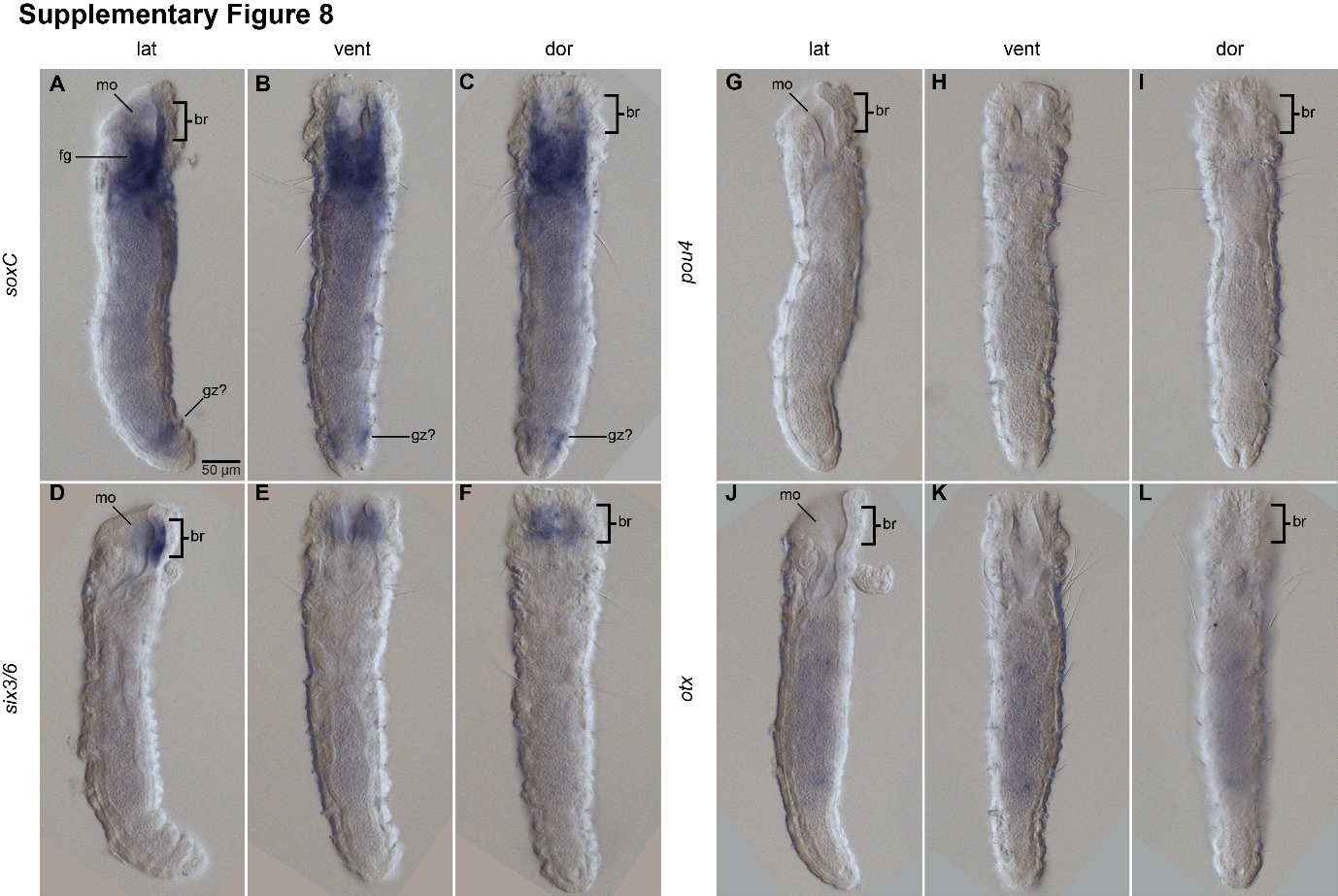

Supplement: Supplementary file 8 — Additional file 8: Supplementary Fig. 8. Neural development in juveniles. DIC images showing expression of soxC, pou4, six3/6 and otx. a, d, g, j Lateral views; b, e, h, k ventral views; c, f, i, l dorsal views. a–csoxC and d–f six3/6 are expressed in the brain (br). g–i pou 4 and j–l otx have no longer any neural expression. a–csoxC is expressed in the foregut (fg), and in the putative growth zone (gz). br: brain; fg: foregut; gz: growth zone; mo: mouth. [file 13064_2024_180_MOESM8_ESM.docx]
