# Supplementary material for: The development of the adult nervous system in the annelid Owenia fusiformis
Source: Neural Dev. 2024 Feb 21;19:3. doi: 10.1186/s13064-024-00180-8 (PMC10880339; doi:10.1186/s13064-024-00180-8)

Additional File 9: Supplementary Figure 9 Neuropeptide-lir elements in the adults. CLSM images of neuropeptide-lir close ups of images in Figure 9. **a–b, e–f, i–j, m–n** ventral views; **c–d, g–h, k–l**, **o–p** dorsal views. **a, e, i, m** Views of the eye showing FVamide-lir, RYamide-lir and MIP-lir, antero-lateral (als) and postero-lateral (pls) somata. **b**, **f**, **j**, **n** Lateral head neurites (lhn) extend toward the tentacles and the trunk. **c**, **g**, **k**, **o** Longitudinal dorsal nerve cord (dnc). **d**, **h**, **l**, **p** Brain ring with associated neurites (ne) and somata (so). als: anterior-lateral somata; br: brain; dnc: dorsal nerve cord; ey: eye; lhn: lateral head neurites; lmc: lateral medullary cord; ne: neurite; pls: posterior-lateral somata; so: somata; tp: tentacle plexus.


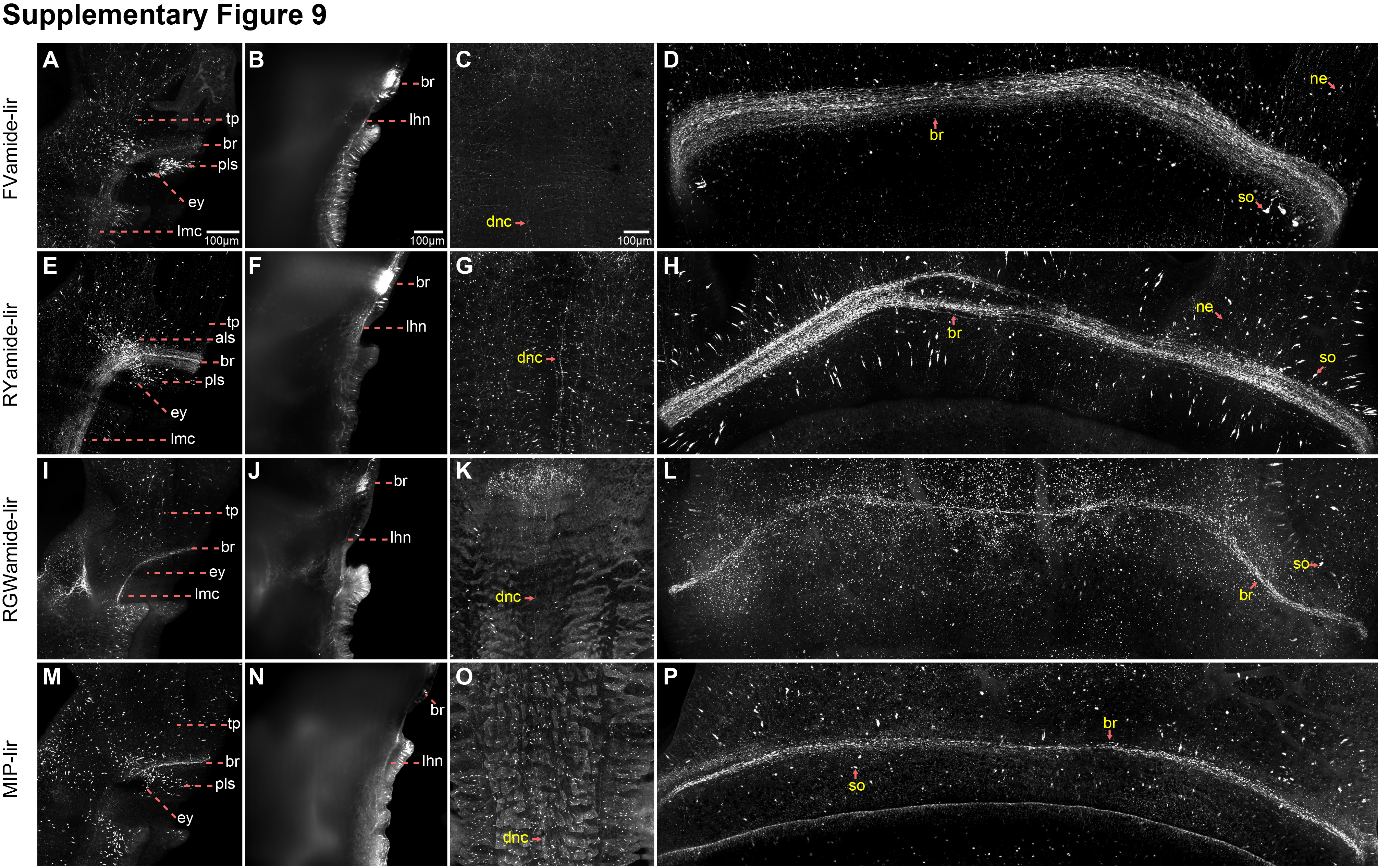

Supplement: Supplementary file 9 — Additional file 9: Supplementary Fig. 9. Neuropeptide-lir elements in the adults. CLSM images of neuropeptide-lir close ups of images in Fig. 9. a–b, e–f, i–j, m–n ventral views; c–d, g–h, k–l, o–p dorsal views. a, e, i, m Views of the eye showing FVamide-lir, RYamide-lir and MIP-lir, antero-lateral (als) and postero-lateral (pls) somata. b, f, j, n Lateral head neurites (lhn) extend toward the tentacles and the trunk. c, g, k, o Longitudinal dorsal nerve cord (dnc). d, h, l, p Brain ring with associated neurites (ne) and somata (so). als: anterior-lateral somata; br: brain; dnc: dorsal nerve cord; ey: eye; lhn: lateral head neurites; lmc: lateral medullary cord; ne: neurite; pls: posterior-lateral somata; so: somata; tp: tentacle plexus. [file 13064_2024_180_MOESM9_ESM.docx]
