# Supplementary material for: The development of the adult nervous system in the annelid Owenia fusiformis
Source: Neural Dev. 2024 Feb 21;19:3. doi: 10.1186/s13064-024-00180-8 (PMC10880339; doi:10.1186/s13064-024-00180-8)
Supplement: Supplementary file 10 — Additional file 10: Supplementary Table 1. Immunoreactivity of neuropeptide and gene expression during the neurogenesis of several annelid species. [file 13064_2024_180_MOESM10_ESM.docx]

Additional File 10: Supplementary Table 1 Immunoreactivity of neuropeptide and gene expression during the neurogenesis of several annelid species.

| Species | Stage | FV-lir | RY-lir | RGW-lir | MIP-lir | *soxC* | *pou4* | *six3/6* | *nk2.1* | *otx* | *ChAt* | Ref. |
| --- | --- | --- | --- | --- | --- | --- | --- | --- | --- | --- | --- | --- |
| *Owenia fusiformis* | 24h larva | ao; lpn | ao, pt, lpn | ao | ao, fn | ao; cells posterior to the mouth | ao | ao | Dorsal part of fg | pt, fg | Cell above fg | (Martín-Durán et al, 2016, 2018, Seudre et al. 2022; this study) |
|  | 3-week larva | ao, jnc | ao, jnc | ao | ao, jnc | br, jr | br, jr | br | br | pt, fg | br, jr | (This study) |
|  | Juv | br, vnc | br, vnc | br, vnc | br, vnc | br, fg, gz | NE | br | fg, gz? | NE | jnc | (Martín-Durán et al., 2018ñ this study) |
|  | Adult | br | br | br | br, ln | - | - | - | - | - | - | This study |
| *Platynereis dumerilii* | 22h-72h |  |  |  | br | vnc | - | br | br | pt, mo | vnc | (Denes et al., 2007; Kerner et al. 2009ñ Tessmar-Raible et al., 2007) |
| *Capitella teleta* | st4 st6  st7 | br np, vnc | br np, vnc | br np, vnc | br np | - | - | - | br | br | - | (Boyle et al. 2014) |
| *Trilobodrilus axi* | Adult | br | - | - | br | - | - | - | - | - | - | (Kerbl et al. 2017) |
| *Dinophilus taeniatus* | Adult | br, vnc, sns | - | - | br, vnc, sns | - |  | - | - | - | - | (Kerbl et al. 2017) |
| *Dimorphilus gyrociliatus* | Adult | br, vnc, sns | - | br | br, sns | - | - | Brain: cell bodies surrounding neuropil. | Brain: anterio-ventral part of the brain | Brain: postero-lateral parts of the brain | - | (Kerbl et al. 2016) |
| *Malacocerus fuliginosus* | 14h-24h larva | - | - | - | - | Anterior domain of episphere | Posterior neuron | - | - | - | - | (Kumar et al. 2020) |
| *Hydroides elegans* | 24 h larva | - | - | - | - | - | - | - | - | Below and dorsal of prototroch | - | (Arenas-Mena and Wong, 2007) |

Ao: apical organ; br: brain; fg: foregut; gz: growth zone; jnc: juvenile nerve cords; jr: juvenile rudiment; ln: lateral nerves; lpn: larval peripheral nerves; mo: mouth; NE: no expression; np: neuropil; pr: prototrochal ring; pt: prototroch; sns: stomatogastric nervous system; vnc: ventral nerve cord.

**Supplementary References**

Arenas-Mena, C., Wong, K.SY. *HeOtx* expression in an indirectly developing polychaete correlates with gastrulation by invagination. *Dev Genes Evol* 217, 373–384 (2007).

Arendt, D., et al. Evolution of the bilaterian larval foregut. *Nature* 409.6816 (2001): 81-85.

Denes, A. S., et al. Molecular architecture of annelid nerve cord supports common origin of nervous system centralization in bilateria. *Cell* 129.2 (2007): 277-288.

Kerbl, A., et al*.* Molecular regionalization in the compact brain of the meiofaunal annelid *Dinophilus gyrociliatus* (Dinophilidae). *EvoDevo* 7, 20 (2016).

Kerbl, A., et al. High diversity in neuropeptide immunoreactivity patterns among three closely related species of Dinophilidae (Annelida). *Journal of Comparative Neurology* 525.17 (2017): 3596-3635.

Kerner, P., et al. Orthologs of key vertebrate neural genes are expressed during neurogenesis in the annelid *Platynereis dumerilii*. *Evolution & development* 11.5 (2009): 513-524.

Kumar, S., et al*.* The development of early pioneer neurons in the annelid *Malacoceros fuliginosus*. *BMC Evol Biol* 20, 117 (2020).

Steinmetz, P.R., et al*.* *Six3* demarcates the anterior-most developing brain region in bilaterian animals. *EvoDevo* 1, 14 (2010).

Steinmetz, P.R.H., et al. The segmental pattern of otx, gbx, and Hox genes in the annelid *Platynereis dumerilii*. *Evolution & development* 13.1 (2011): 72-79.

Tessmar-Raible, K., et al. Conserved sensory-neurosecretory cell types in annelid and fish forebrain: insights into hypothalamus evolution. *Cell* 129.7 (2007): 1389-1400.
